# Cost-effectiveness analysis of point-of-care tests for causes of non-malarial febrile illnesses in low-resource settings: a case study from Lao PDR

**DOI:** 10.1136/bmjph-2025-003523

**Published:** 2026-06-22

**Authors:** Chris Painter, Koukeo Phommasone, Mayfong Mayxay, Elizabeth A Ashley

**Affiliations:** 1 Lao-Oxford-Mahosot Hospital-Wellcome Trust Research Unit (LOMWRU), Microbiology Laboratory, Mahosot Hospital, Vientiane, Lao People’s Democratic Republic; 2 University of Oxford Centre for Tropical Medicine and Global Health, Oxford, UK; 3 Mahidol-Oxford Tropical Medicine Research Unit, Bangkok, Thailand; 4 Unit for Health Evidence and Policy, University of Health Sciences, Ministry of Health, Vientiane, Lao People’s Democratic Republic; 5 Institute of Research and Education Development, University of Health Sciences, Vientiane, Lao People’s Democratic Republic

**Keywords:** economics, Public Health, Public Health Practice

## Abstract

**Introduction:**

In many low- and middle-income countries, there is widespread use of point-of-care tests (POCTs) to diagnose a variety of infectious diseases, even in hospitals due to weak laboratory capacity. Often, POCT use is unregulated and expensive while the use of substandard tests is common. A cost-effectiveness analysis was conducted to investigate which POCTs for non-malarial febrile illness (NMFI) represent the best use of resources in Lao PDR in outpatient settings, assuming best-in-class POCTs are used.

**Methods:**

A decision-tree cost-effectiveness analysis was conducted to simulate use of POCTs of interest for outpatients presenting with NMFI, compared to clinical assessment alone. The analyses were performed from both healthcare provider’s perspective and a limited societal perspective, which included cost estimates of the contribution of antibiotic consumption by class to future AMR costs. Deterministic and probabilistic analyses were performed.

**Results:**

In probabilistic and deterministic analyses, dengue and rickettsia POCTs were consistently cost-effective and the most cost-effective of the four POCTs evaluated. Deterministic analyses estimated that typhoid and leptospirosis POCTs were cost-effective compared with clinical assessment alone. However, in probabilistic analyses, clinical assessment was more likely to be cost-effective than typhoid or leptospirosis POCTs at the estimated cost-effectiveness threshold in the Lao PDR.

**Conclusions:**

This evaluation was able to make use of comprehensive primary data from fever studies in Lao PDR, giving an understanding of the aetiology and prescribing behaviour of physicians for undifferentiated NMFI. However, sensitivity analyses showed that there was substantial uncertainty about which strategy would be most cost-effective and if substandard POCTs were analysed then it is more likely that clinical assessment alone would be the most cost-effective strategy. This research shows that there is good reason to be cautious about the overuse of POCTs for NMFI and whether they represent good value for money in low-income contexts compared with clinical assessment alone.

**WHAT IS ALREADY KNOWN ON THIS TOPIC**

- Non-malarial febrile illness (NMFI) in Lao PDR can be due to various underlying causes; some causes require treatment with antibiotics.
- In low- and middle-income countries (LMICs) like Lao PDR, point-of-care test (POCT) use to aid diagnosis is often unregulated and expensive, while use of substandard tests is common.
- Concerns around antimicrobial resistance have led to the implementation of POCTs for a range of causes, but economic evidence to support their use in LMICs is sparse.

**WHAT THIS STUDY ADDS**

- This research used a novel framework for evaluating the cost-effectiveness of POCTs for febrile illness, a clinical presentation with a multitude of underlying causes.

**HOW THIS STUDY MIGHT AFFECT RESEARCH, PRACTICE OR POLICY**

- This research shows that there is good reason to be cautious about the overuse of POCTs for NMFI and whether they represent good value for money in low-income contexts compared with clinical assessment alone.

## Background

 Point-of-care tests (POCTs) enable rapid, accurate diagnosis and targeted treatment of infectious and other diseases. They are also used for disease surveillance and for infection prevention and control, as shown by the large-scale adoption of COVID-19 rapid diagnostic tests worldwide during the pandemic. In many low- and middle-income countries (LMICs), there is widespread use of POCTs to diagnose a variety of infectious diseases, even in hospitals, due to weak laboratory capacity. Often, POCT use is not regulated and use of substandard tests is common.^1^ The situation has improved for selected diseases which have vertical programmes such as HIV and malaria, following increased scrutiny of the quality of POCTs by donors such as the Global Fund and the introduction of the WHO prequalification scheme for in vitro diagnostic tests.^2^

Malaria has gone down in many areas, but fever remains a leading reason for healthcare seeking in tropical countries. Introduction of POCTs to diagnose malaria has had the unwanted effect of driving up antibiotic use to treat patients presenting with non-malarial febrile illness (NMFI), most of whom would have received chloroquine 20 years ago.^3^ Antibiotic use is considered one of the most important drivers for the current global crisis of bacterial antimicrobial resistance (AMR), associated with an estimated 4.71 million (4.23–5.19) deaths in 2021.^4^ The role of diagnostic tests as a health technology capable of controlling AMR is a priority area for evaluation, although scientific evidence to guide many policy interventions to tackle AMR in LMICs is weak at present.^5 6^ A multicentre comparative study of the impact of introducing a package of POCTs for the management of febrile illness showed mixed results, with no impact at the sites in Uganda but a moderate reduction in antimicrobial prescribing in Ghana and Burkina Faso (risk difference 11%–17%), especially in patients with respiratory symptoms.^7^,^14^

In Lao PDR, a lower-middle-income country in Southeast Asia, with a population of 7.2 million people, data from point-prevalence surveys in central and provincial hospitals have shown that around 70% of all inpatients are prescribed antimicrobials, which is one of the highest rates in the world.^15^ Rates of AMR are also increasing, particularly extended-spectrum beta-lactamase producing *Escherichia coli* which now accounts for 50% of all *E. coli* bloodstream infections.^16^ Malaria in Lao PDR is now restricted to a few southern provinces; however, fever remains a leading cause for healthcare consultation. Studies have shown the most common aetiologies of febrile illness are dengue, rickettsial diseases, leptospirosis, typhoid fever and respiratory viruses.^17^,^19^ The majority of hospitals in Lao PDR use POCTs to diagnose some of these infections; however, their use is unregulated and for some, the performance of the tests is uncertain or unverifiable. Health technology assessment (HTA) methods can help health systems to use their scarce resources more efficiently, by systematically evaluating the properties, benefits and costs of health technologies and services. HTA methods are increasingly used in the Southeast Asian region^20^ and are in the early stages of being adopted as part of the policy decision-making process in Lao PDR where a new Unit for Health Evidence and Policy was created in 2021. A cost-effectiveness analysis was conducted to investigate which POCTs for NMFI represent the best use of resources in Lao PDR in outpatient settings.

## Methods

A cost-effectiveness analysis was conducted to evaluate the use of the POCTs of interest for outpatients presenting with NMFI. A variety of POCT strategies were compared, including in isolation and combination, as well as diagnostic strategies without the use of POCTs. Reporting of the model has been conducted in accordance with the Consolidated Health Economic Evaluation Reporting Standards (CHEERS) checklist, which can be found in the online supplemental materials. This was a secondary data analysis and modelling study using anonymised aggregated data from two studies of the causes of fever (Expanded Fever Surveillance (EFS) and Febrile Illness in a Broad Range of Endemicities (FIEBRE)).

### Model structure

**Figure 1:**
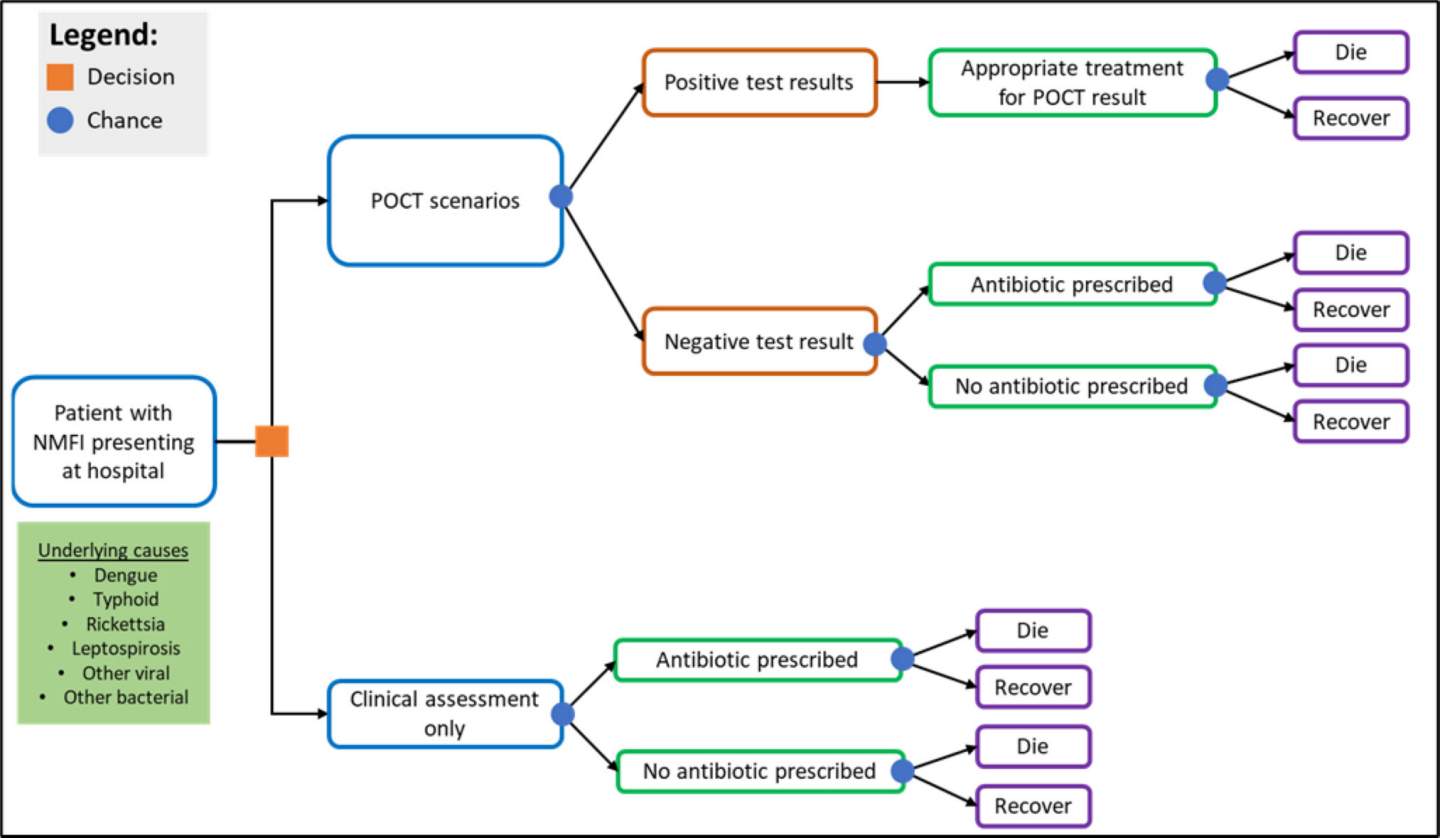
Decision tree structure. NMFI, non-malarial febrile illness; POCT, point-of-care test.

A simple decision tree model was developed to simulate the diagnostic process for NMFI outpatients in Lao PDR (figure 1). The model used the prevalences of the different causes of NMFI and antibiotic prescribing rates from primary data in Lao PDR, with the diagnostic accuracy of best-in-class POCTs for NMFI causes. In the clinical assessment arm, it was assumed that no POCTs were used and the physician would determine whether to prescribe an antibiotic or not. In the four POCT arms (for dengue, typhoid, rickettsia and leptospirosis), physicians assumed positive test results were true positives and prescribed accordingly. If the test result was negative, the physician prescribed according to clinical assessment. POCTs were evaluated individually, and joint or sequential testing was not considered in the analysis.

The analyses were performed from both the healthcare provider’s perspective and a limited societal perspective, which included cost estimates of the contribution of antibiotic consumption by class to future AMR costs. The analyses were conducted over a lifetime time horizon due to the presence of mortality in the model. Discounting was not applied as all costs occurred in the first year.

As with other economic analyses of this type,^21 22^ it was assumed that following the infection episode, patients either died or fully recovered, and did not have long-term health impacts associated with the infection.

### Model parameters

Primary data from the EFS study and Lao PDR cohort of the FIEBRE studies were used.^23^ The data used included the identified causes of febrile illness as well as the corresponding treatments prescribed. The proportions of confirmed and probable diagnoses of all patients were used as the base case estimates for the prevalence of the causes, as in Lubell *et al.*^22^ Antibiotic prescribing rates by underlying cause were used to simulate the effectiveness of clinical assessment alone in practice. This was assumed to be an appropriate proxy because clinicians did not usually have access to the laboratory analysed diagnostic result for the patient before prescribing in both the EFS and FIEBRE studies.

POCT efficacy was obtained from targeted literature searches of PubMed and Google Scholar to obtain best-in-class sensitivity and specificity performance of POCTs for dengue, leptospirosis, rickettsia and typhoid. POCT costs were assumed to be US$2 per test to provide indicative results in the base case. Key model parameters are listed in table 1.

**Table 1:** Key model parameters

Model parameter	Value	Source
Antibiotic prescribing rate: clinical assessment alone		
Dengue	69.0%	EFS study data (confirmed dengue diagnoses)
Typhoid	91.9%	Pooled EFS study data (confirmed diagnoses) with FIEBRE study, adults and children (percentage of confirmed and probable cases on any antibiotics)^23^
Rickettsia	76.2%	
Leptospirosis	68.0%	
Other viral	10.7%	
Other bacterial	91.5%	
POCT test performance		
Dengue	Sensitivity: 82.7% (72.2%–94.3%) Specificity: 100% (94.3%–100%)	Lee *et al*^38^ NS1+IgG+IgM. SD test
Typhoid	Sensitivity: 93.0% (87.0%–97.0%) Specificity: 89.0% (85.0%–93.0%)	Munira *et al*^39^
Rickettsia	Sensitivity: 92.1% (84.0%–97.0%) Specificity: 96.1% (88.0%–98.0%)	Kim *et al*^40^ ImmuneMed
Leptospirosis	Sensitivity: 78.0% (71.0%–83.0%) Specificity: 98.0% (97.0%–98.0%)	Goris *et al*^41^ Leptocheck WB
Patient characteristics		
Average age	28	FIEBRE Lao PDR cohort data^23^
Proportion of adolescents and adults	72.5%	
Proportion of children	27.5%	
Adult weight (kg)	56.6	WorldData^42^
Child weight (kg)	14.6	Pooled SEANUTS average^43^
Fever causes		
Dengue	22.5%	FIEBRE Lao PDR cohort data^23^ Restricted to confirmed and probable diagnoses only (inpatients and outpatients)
Typhoid	1.1%	
Rickettsia	5.1%	
Leptospirosis	6.3%	
Other viral	60.7%	
Other bacterial	4.4%	
Cost per course prescribed—adults (US$)		
Amoxicillin	$0.42	Management Sciences for Health, International Medical Products Price Guide 2015 edition^44^ Inflated to 2024 values
Ampicillin	$8.87	
Azithromycin	$1.58	
Ceftriaxone	$6.80	
Doxycycline	$0.27	
Ofloxacin	$2.14	
Cost per course prescribed—children (US$)		
Amoxicillin	$0.33	Management Sciences for Health, International Medical Products Price Guide 2015 edition^44^ Inflated to 2024 values
Ampicillin	$5.17	
Azithromycin	$0.56	
Ceftriaxone	$5.53	
Ofloxacin	$0.55	
Hospitalisation costs (US$)		
Inpatient bed day cost	$9.88	WHO Choice tertiary hospital Lao PDR values reverted from PPP estimates^45^ Inflated to 2024 values
Outpatient bed day cost	$1.94	WHO choice tertiary hospital Lao PDR values reverted from PPP estimates^45^ Inflated to 2024 values
Proportion of patients hospitalised		
Dengue	13.6% (12.0%–15.0%)	Vong *et al*^46^
Typhoid (diagnosed+treated)	6% (0.4%–25%)	Bilcke *et al*^47^
Typhoid (undiagnosed)	12% (0.8%–50%)	Assumed doubled hospitalisation risk compared with diagnosed and appropriately treated typhoid
Mortality risk		
Typhoid (untreated)	1% (0.4%–4%)	Mogasale *et al*^48^
Rickettsia (untreated)	6% (0%–12.5%)	Bonell *et al*^49^ Range adjusted by expert opinion but informed by Swe^50^
Leptospirosis (untreated)	3% (0%–10.0%)	Swe^24^ Range adjusted by expert opinion
Dengue (unmonitored)	2.5% (1.0%–5.0%)	WHO, Regional Office for South-East Asia^51^ Range taken from Ranjit *et al* (1.0%–5.0%)^52^
Dengue (monitored)	1% (0%–2.0%)	Ranjit *et al*^52^ Assumed range of 0%–2.0%
Disability weights		
Non-hospitalised typhoid	0.051 (0.032–0.074)	IHME Global Burden of Disease 2019 Disability Weights^53^ Acute typhoid infection
Hospitalised typhoid	0.133 (0.088–0.190)	IHME Global Burden of Disease 2019 Disability Weights^53^ Severe typhoid fever
Duration of illness (days)		
Dengue (total, hospitalised)	3.2 (0.65–5.75)	Senavong *et al*^54^
Typhoid (total)	14 (7–21)	Assumption based on NHS^55^
Typhoid (hospitalisation period)	6 (3–9)	Bilcke *et al*^47^

EFS, Extended Fever Surveillance; FIEBRE, Febrile Illness Evaluation in a Broad Range of Endemicities; IHME, Institute for Health Metrics and Evaluations; NHS, National Health Service; NS1, non-structural protein 1; PDR, People’s Democratic Republic; POCT, point-of-care test; PPP, Purchasing power parity; SEANUTS, South East Asian Nutrition Surveys; USD, US dollars.

An approach to modelling treatment efficacy of antibiotics for causes of febrile illness was adapted from Swe.^24^ Parameters for antibiotic treatment efficacy vs the bacterial causes (typhoid, rickettsia and leptospirosis) were obtained from Swe and supplemented by targeted literature searches and expert opinion, these are detailed in online supplemental table 1.^25 26^ The classes of antibiotics prescribed and the societal costs arising from AMR are detailed in online supplemental tables 2 and 3.

### Outcomes

Cost outcomes were measured in 2024 US dollars, while health outcomes were quantified in terms of disability-adjusted life years (DALYs). A deterministic sensitivity analysis (DSA) was conducted to identify the key drivers of cost-effectiveness for the diagnostic strategies. A probabilistic sensitivity analysis (PSA) was used to understand the impact of the collective uncertainty in the economic evaluation, and also to predict the likelihood of each strategy proving cost-effective at a range of willingness-to-pay (WTP) thresholds, depicted on a cost-effectiveness acceptability curve. The base case WTP for Laos was taken from Woods *et al*,^27^ and inflated to a 2024 value of US$1029/DALY averted. Parameters were assigned appropriate distributions: beta for probabilities, gamma for costs and Dirichlet for disease prevalences. Correlations between parameters were not included due to a lack of data. Scenario analyses explored the cost-effectiveness of the strategies at various levels of disease prevalence, which could aid decision makers as new data on undifferentiated febrile illnesses in Lao PDR becomes available in the future. Threshold analyses were conducted to understand the purchase price at which the POCTs could prove cost-effective.

### Prevalence scenario and sensitivity analyses

Given the range of underlying causes of febrile illness and the competing diagnostic targets as interventions, a framework was developed to more comprehensively consider the uncertainty in the model while systematically altering the aetiological prevalences. The four underlying causes targeted by the diagnostics (dengue, typhoid, rickettsia and leptospirosis) were altered from 0% to 60% in 0.1% increments, with the other causes adjusted upwards or downwards while maintaining their relative contributions. The deterministic net monetary benefits (NMBs) compared with clinical assessment alone were recorded at each increment. Furthermore, this scenario was then repeated in 0.5% increments, with the PSA reperformed at each increment (with the new prevalence values fixed) to incorporate the combined uncertainty in the model at each prevalence value. The results of the iterative PSA were then recorded along with 95% CIs.

## Results

### Main results

The deterministic results suggested that all of the POCTs could be cost-effective in the base case compared with clinical assessment alone, at a cost of US$2 per test. The deterministic results are displayed in a cost-effectiveness plane shown in online supplemental figure 1. The most cost-effective POCT (ie, lowest incremental cost-effectiveness ratio) compared with clinical assessment was for dengue, followed by rickettsia, typhoid and finally leptospirosis. However, the NMB of the rickettsia POCT was greater than that for dengue.

However, the estimates of cost-effectiveness were at odds with the probabilistic results which took into account the combined uncertainty in the model. The results of the PSA are displayed in a scatter plot on the cost-effectiveness plane (online supplemental figure 6). The PSA results show the considerable uncertainty in the cost-effectiveness of the POCTs, likely due to large uncertainty of some of the key parameters like mortality for untreated conditions. The mean probabilistic results are shown in table 2. Similar to the deterministic results, the dengue and rickettsia POCTs remained the most cost-effective, while typhoid and leptospirosis POCTs also remained cost-effective in the estimates. As the scatter plot shows, there were simulations in which the dengue POCT was cost-saving due to the potential of averting unnecessary antibiotic prescriptions with a positive result. These results were consistent from both the healthcare provider and societal perspectives. With regards to antimicrobial stewardship, only the dengue POCT was estimated to reduce antibiotic prescribing, while the other POCTs were estimated to increase both antibiotic prescribing and inappropriate prescriptions of antibiotics.

**Table 2:** Mean probabilistic cost-effectiveness results

Intervention	Total costs (95% CI) (US$)	Total DALYs (95% CI)
Clinical assessment alone	4.96 (4.80 to 5.13)	0.4005 (0.3659 to 0.4352)
Dengue POCT	6.08 (5.93 to 6.23)	0.2795 (0.2489 to 0.3100)
Typhoid POCT	8.01 (7.82 to 8.20)	0.3554 (0.3230 to 0.3878)
Rickettsia POCT	7.24 (7.07 to 7.41)	0.2774 (0.2564 to 0.2984)
Leptospirosis POCT	7.16 (6.99 to 7.34)	0.3687 (0.3382 to 0.3992)

Incremental results versus clinical assessment			
Intervention	Mean incremental cost (95% CI (US$)	Mean incremental DALYs averted (95% CI)	Mean incremental cost-effectiveness ratio (95% CI)
Dengue POCT	1.12 (1.11 to 1.13)	0.1211 (0.1169 to 0.1252)	$9.25/DALY averted (8.87 to 9.67)
Typhoid POCT	3.05 (3.03 to 3.07)	0.0451 (0.0428 to 0.0474)	$67.63/DALY averted (63.92 to 71.73)
Rickettsia POCT	2.28 (2.27 to 2.28)	0.1232 (0.1095 to 0.1368)	$18.52/DALY averted (16.66 to 20.75)
Leptospirosis POCT	1.96 (1.93 to 1.99)	0.0341 (0.0310 to 0.0371)	$57.47/DALY averted (53.10 to 61.96)

DALYs, disability-adjusted life years; POCT, point-of-care test.

**Figure 2:**
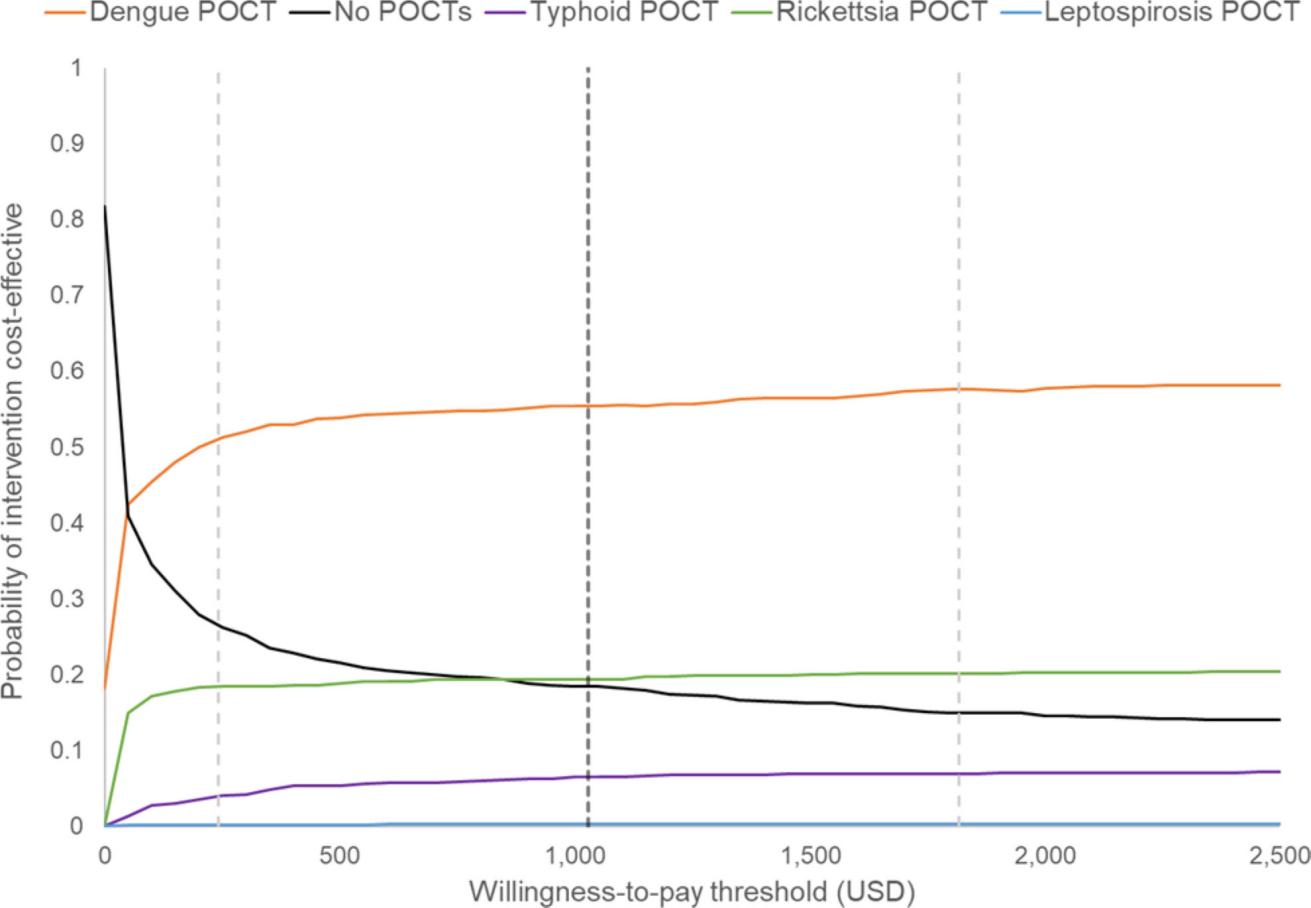
Cost-effectiveness acceptability curve. Dashed grey lines indicate the upper and lower bounds of the estimated willingness-to-pay thresholds, while the thicker dotted black line indicates the mean value of the willingness-to-pay threshold. POCT, point-of-care test; USD, US dollars.

The cost-effectiveness acceptability curve in figure 2 shows the complexity in understanding which intervention is the most cost-effective approach. As the WTP threshold increases, dengue and rickettsia POCTs become the two most likely to be cost-effective interventions. However, no strategy exceeds a 60% likelihood of cost-effectiveness apart from the clinical assessment strategy at very low WTP thresholds. At all WTP thresholds, the typhoid and leptospirosis POCTs were very unlikely to be the most cost-effective strategy.

### Further analyses

**Figure 3:**
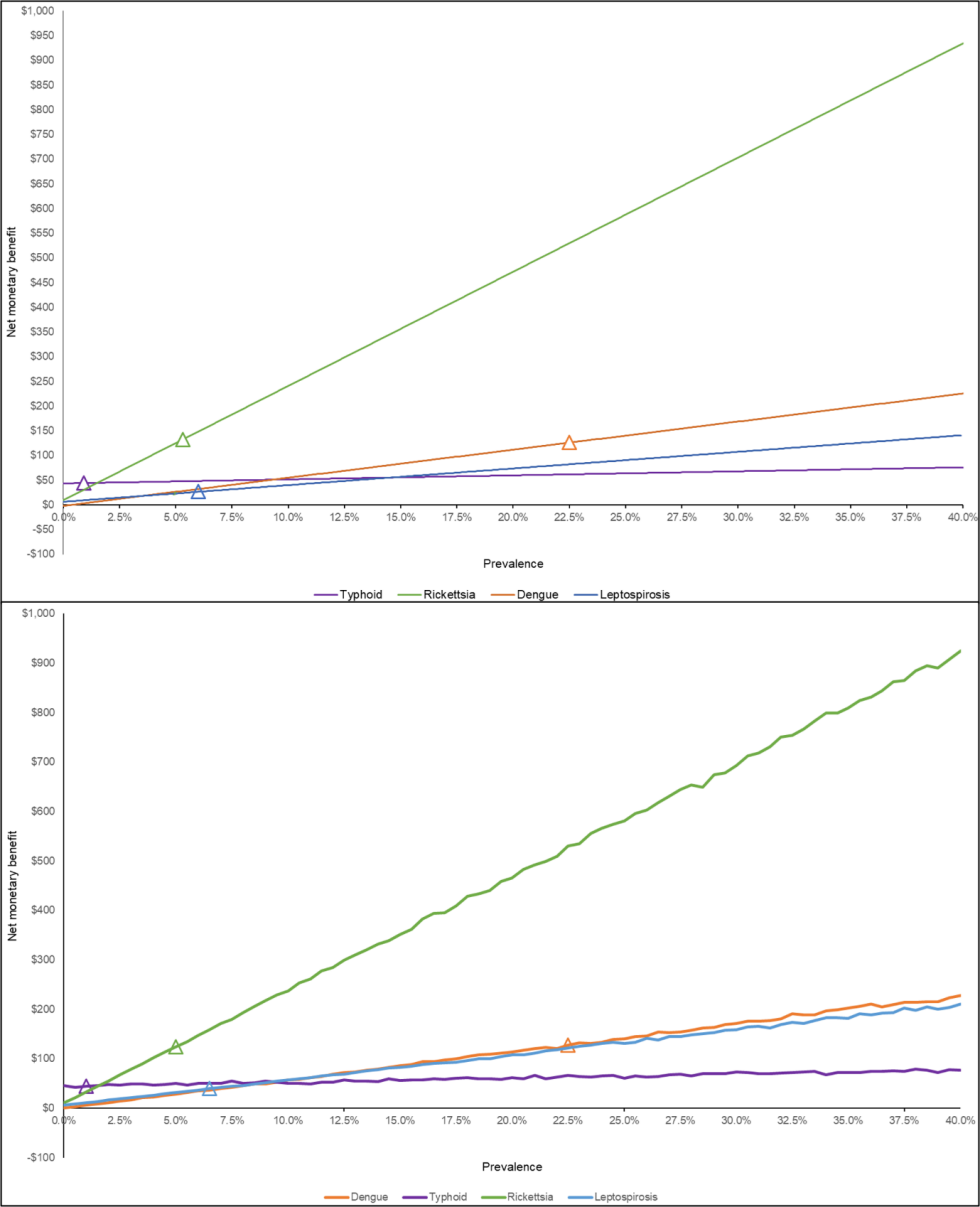
Top: deterministic epidemiological scenario analysis. Bottom: probabilistic epidemiological scenario analysis.

The results of a scenario where the prevalence of underlying causes of febrile illness was varied displayed the influence of these parameters on the cost-effectiveness of the corresponding POCTs (figure 3). These results display the sensitivity of the cost-effectiveness results to changes in the prevalence of the underlying causes. As expected, all POCTs were more cost-effective when the prevalence of the corresponding condition increased, as more people could benefit from an accurate diagnosis. In the probabilistic prevalence analysis all of the POCTs were less cost-effective than in the deterministic analysis, with the dengue POCT the most impacted strategy. During the data collection period for the FIEBRE study, there was an unusually high incidence of dengue in Lao PDR, and these results indicated how the cost-effectiveness of the dengue POCT would be reduced in years of reduced dengue incidence. Further probabilistic analyses are displayed in online supplemental figures 7–10 with 95% CIs.

Triangles denote the prevalence values used in the base case deterministic analyses observed in the FIEBRE study.

The results of the DSAs are displayed in online supplemental figures 2–5. Predictably, the model parameters which were most influential, in terms of their impact on cost-effectiveness, were the mortality of untreated rickettsia and leptospirosis and the effectiveness of clinical assessment in the absence of POCTs. Underlying cause prevalences were not included in the DSA because they cannot be individually altered without adjusting the other causes, so they are not present in the tornado plot.

## Discussion

A recent systematic review summarised the literature of economic evaluations of point-of-care diagnostics impacting AMR and antimicrobial usage, the study found that most of the evaluations were focused on CRP tests, while five of the studies were conducted in LMICs and only three studies were conducted in Southeast Asia.^28^ Therefore, this research adds to this limited body of literature and directly compares four POCTs that could be deployed for the diagnosis of the same clinical presentation.

This manuscript has provided a novel framework for evaluating the cost-effectiveness of POCTs for febrile illness, a clinical presentation with a multitude of underlying causes. Given this variation, systematic and extensive interrogation of the cost-effectiveness at varied prevalences of underlying causes is necessary to understand the best use case for these tests, particularly as even within countries, the incidence of causes of NMFI vary between regions and seasonally. The previously mentioned systematic review stated that a key limitation in previous economic evaluations of this area was that real-world evidence was rarely available or used. This evaluation was able to make use of comprehensive primary data from the FIEBRE and EFS fever studies in Lao PDR,^23^ giving an understanding of the aetiology and prescribing behaviour of physicians for undifferentiated NMFI. However, despite the strengths of this data, there were still important data gaps that meant assumptions were required, particularly with regards to quantifying the efficacy of antibiotics with treating individual causes of fever and inappropriate treatment.^29^ Similarly, although published mortality estimates for treated and untreated febrile illness were used in the model, reliable estimates for these are hard to obtain (eg, due to ethical issues) and were not specific to Lao PDR.

Concerns around AMR have led to the implementation of POCTs for a range of causes, but economic evidence to support their use in LMICs is sparse.^28 30^ The POCTs have a non-trivial cost and in resource-limited settings it is important to understand the extent to which these tests are economically efficient, and also to be able to choose between the tests where necessary. The findings of this research are applicable to many LMICs endemic with dengue, typhoid, rickettsia and leptospirosis. Even if the contribution of the individual causes of the fevers is different to that estimated in Lao PDR, the results can be interpreted for a range of other epidemiological environments. Policymakers should consider that the cost-effectiveness analyses were conducted assuming that best-in-class POCTs were being used, in many settings (including LMICs), it is common to use alternative brands of test that may perform more poorly. In such cases, decision-makers should consider that substandard POCTs will be even less cost-effective than is indicated in these analyses. This assumption is particularly pertinent for the analysis of the rickettsia POCT. It was assumed in the model that the POCT was equally accurate across rickettsias, however in practice many POCTs perform better in either scrub typhus or typhus group rickettsiae. As such, the cost-effectiveness of the rickettsia POCT would be reduced in these circumstances. Though figure 3 shows that even if the rickettsia POCT was only accurate in one of the subtypes, the rickettsia POCT would likely still be the second most cost-effective POCT.

It is also worth considering that the accuracy of clinical assessment without POCTs is modifiable, for example in the case of scrub typhus rates of eschar (a skin lesion pathognomonic of scrub typhus) detection vary substantially by country and a recent review reported a prevalence or detection rate in Laos PDR as 32.6%, well below the average of 58.0% and the highest reported rates which were above 80%.^31^ Furthermore, use of local seasonal endemicity data may also aid the accuracy of clinical assessment which would further increase the cost-effectiveness of clinical assessment and in turn reduce the cost-effectiveness of POCTs.

A limitation of the analysis is that neither joint nor sequential use of multiple POCTs were considered in the model. However, after discussion with clinicians in Lao PDR, the research team decided that in clinical practice doctors and patients would likely not have time to conduct sequential testing in busy clinic settings. Joint testing, perhaps via a multiplex POCT, could be possible but is not currently available and was not considered in this analysis as a result. However, other research has considered the use of multiplex POCT for dengue, CRP and typhoid in other LMIC settings, and found it was potentially cost-effective.^32^ A further limitation is that long-term sequelae from the infections were not considered due to a lack of data to quantitatively substantiate any persistent health impacts in the longer term, as has been noted in other economic analyses.^33 34^ There is a need for better data to facilitate the inclusion of these impacts in future economic analyses.

The results of the analyses paper show that the dengue and rickettsia POCTs were consistently estimated to be the most cost-effective POCTs for NMFI in Lao PDR. Furthermore, outbreaks and periods of low incidence can matter: The prevalence of dengue fever was unusually high (22.5%) in the FIEBRE dataset, and therefore there was a greater modelled capacity to benefit from correct dengue treatment by reduced mortality and unnecessary antibiotic prescribing. In clinical reality, often in outbreak situations confirmation of individual cases is abandoned at a certain prevalence threshold when all patients meeting the case definition are then assumed to have the outbreak disease. At times of lower dengue incidence, the dengue POCT would likely be less cost-effective while the other POCTs may increase in cost-effectiveness if the contribution of their target pathogens has increased as a result. However, it is extremely unlikely that typhoid or leptospirosis POCTs would be the most cost-effective strategy, regardless of the prevalence of the pathogens. The cost-effectiveness of the rickettsia POCT can be explained by rickettsial infections having a comparatively high mortality rate and requiring less frequently prescribed antibiotics to be effectively treated.

Healthcare workers’ attitudes and perceptions of the value of POCTs in low-income settings should also be considered. Healthcare workers are generally in favour of their use, particularly in resolving differential diagnoses and clinical uncertainties,^35^ even when they are concerned about the performance and accuracy of the POCTs.^36^ It is important to consider that there may be additional impacts from using POCTs in clinical practice that were not captured in the economic analysis, for example, if the utilisation of a POCT reduces the time taken by clinicians to manage patients then the reduced staff time cost was not considered. Another consideration outside of the scope of the economic analysis is the additional environmental cost of routinely using disposable POCTs, which typically are made from plastic and imported from outside of Lao PDR, that is not present with clinical assessment alone.

If a POCT must be used, the analysis suggests that either a dengue POCT or a rickettsia POCT that is accurate for both scrub typhus and typhus group should be prioritised over the other POCTs for diagnosing NMFI in Lao PDR, assuming the POCTs are the same price. However, the PSA results showed that there was substantial uncertainty about which strategy would be most cost-effective, and if substandard POCTs were analysed then it is more likely that clinical assessment alone would be the most cost-effective strategy. The probabilistic results reflect the combined uncertainty in the model and therefore should be considered more robust in comparison to the deterministic results, particularly when the results between the two differ substantially.^37^ These results add to previous research in Lao PDR which found that POCTs for febrile illness were unlikely to be cost-effective.^22^ This research shows that there is good reason to be cautious about the overuse of POCTs for NMFI, and whether they represent good value for money in low-income contexts compared with clinical assessment alone.

## Supplementary material

10.1136/bmjph-2025-003523 online supplemental file 1

## Data Availability

All data relevant to the study are included in the article or uploaded as supplementary information.
