# Supplementary material for: Cost-effectiveness analysis of point-of-care tests for causes of non-malarial febrile illnesses in low-resource settings: a case study from Lao PDR
Source: BMJ Public Health. 2026 Jun 22;4(2):e003523. doi: 10.1136/bmjph-2025-003523 (PMC13289369; doi:10.1136/bmjph-2025-003523)
Supplement: online supplemental file 1 [file bmjph-4-2-s001.docx]

# Supplementary Materials

## Additional Model Parameters

Supplementary Table 1. Antibiotic effectiveness parameters

| Parameter | Value | Source |
| --- | --- | --- |
| Amoxicillin and azithromycin | | |
| Rickettsia | 97% (87-99%) | Swe PhD thesis table 5^1^ from Phimda et al. 2007^2^ |
| Leptospirosis | 97% (87-99%) | Swe PhD thesis table 5^1^ from Phimda et al. 2007^2^ |
| Typhoid | 90% (80-99%) | Expert opinion |
| Azithromycin | | |
| Rickettsia | 97% (87-99%) | Swe PhD thesis table 5^1^ from Phimda et al. 2007^2^ |
| Leptospirosis | 97% (87-99%) | Swe PhD thesis table 5^1^ from Phimda et al. 2007^2^ |
| Typhoid | 90% (80-99%) | Expert opinion |
| Ceftriaxone | | |
| Rickettsia | 0% | Arjyal et al. 2016^3^ |
| Leptospirosis | 99% (79.2-99%) | Expert opinion |
| Typhoid | 90% (80-99%) | Expert opinion |
| Doxycycline | | |
| Rickettsia | 97% (79.2-99%) | Swe PhD thesis table 7^1^ from Phimda et al. 2007^2^ |
| Leptospirosis | 97% (79.2-99%) | Swe PhD thesis table 7^1^ from Phimda et al. 2007^2^ |
| Typhoid | 0% | Expert opinion |
| Ofloxacin | | |
| Rickettsia | 20% (14-26%) | Swe PhD thesis table 7^1^ (assumed the same as ciprofloxacin) |
| Leptospirosis | 20% (14-26%) | Swe PhD thesis table 7^1^ (assumed the same as ciprofloxacin) |
| Typhoid | 90% (80-100%) | Expert opinion |
| Penicillins | | |
| Rickettsia | 0% | Swe PhD thesis table 5^1^ |
| Leptospirosis | 99% (90%-99%) | Swe PhD thesis table 5^1^ |
| Typhoid | 0% | Expert opinion |

Ceftriaxone, ofloxacin and azithromycin were assumed to be equally as effective for treating typhoid.^4^

Supplementary Table 2. Antibiotic prescription frequencies from the FIEBRE study

| Antibiotic class | Proportion of prescriptions |
| --- | --- |
| Tetracyclines | 2.8% |
| Fluoroquinolones | 1.7% |
| Macrolides | 3.0% |
| Cephalosporins | 68.7% |
| Broad spectrum penicillins | 13.5% |
| Narrow spectrum penicillins | 10.3% |

Note: No carbapenems as meropenem was not available in Lao PDR at the time of data collection.

Supplementary Table 3. Societal antimicrobial resistance costs due to antibiotic consumption

| Antibiotic | Cost per course | Source |
| --- | --- | --- |
| Ofloxacin | $6.30 | Thailand values inflated to 2023 and then converted to Lao PDR using GDP per capita PPP ratio with Thailand. Shrestha et al. 2018^5^ <https://moru.shinyapps.io/amrcost/> |
| Doxycycline* | $0.89 |  |
| Azithromycin | $0.10 |  |
| Ceftriaxone | $3.15 |  |
| Amoxicillin | $0.89 |  |

*Assumed to be equivalent to narrow spectrum penicillin

## Deterministic Model Results

Supplementary Figure 1. Deterministic cost-effectiveness plane of interventions vs. diagnosis relying on clinical assessment alone


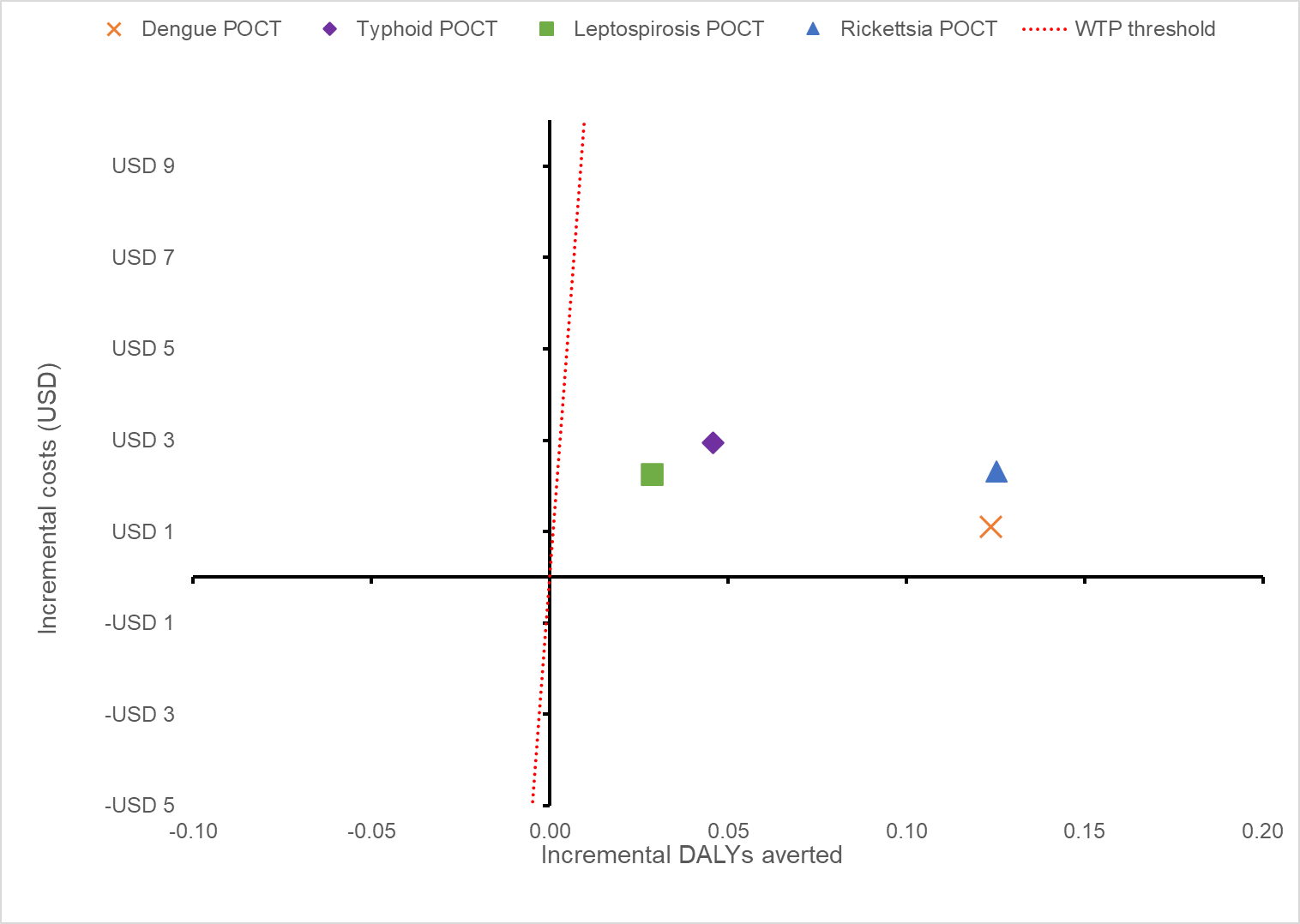


Abbreviations: DALYs, disability-adjusted life years; POCT, point-of-care test; WTP, willingness-to-pay.

## Deterministic Sensitivity Analyses

Supplementary Figure 2. Deterministic sensitivity analysis tornado plot – dengue


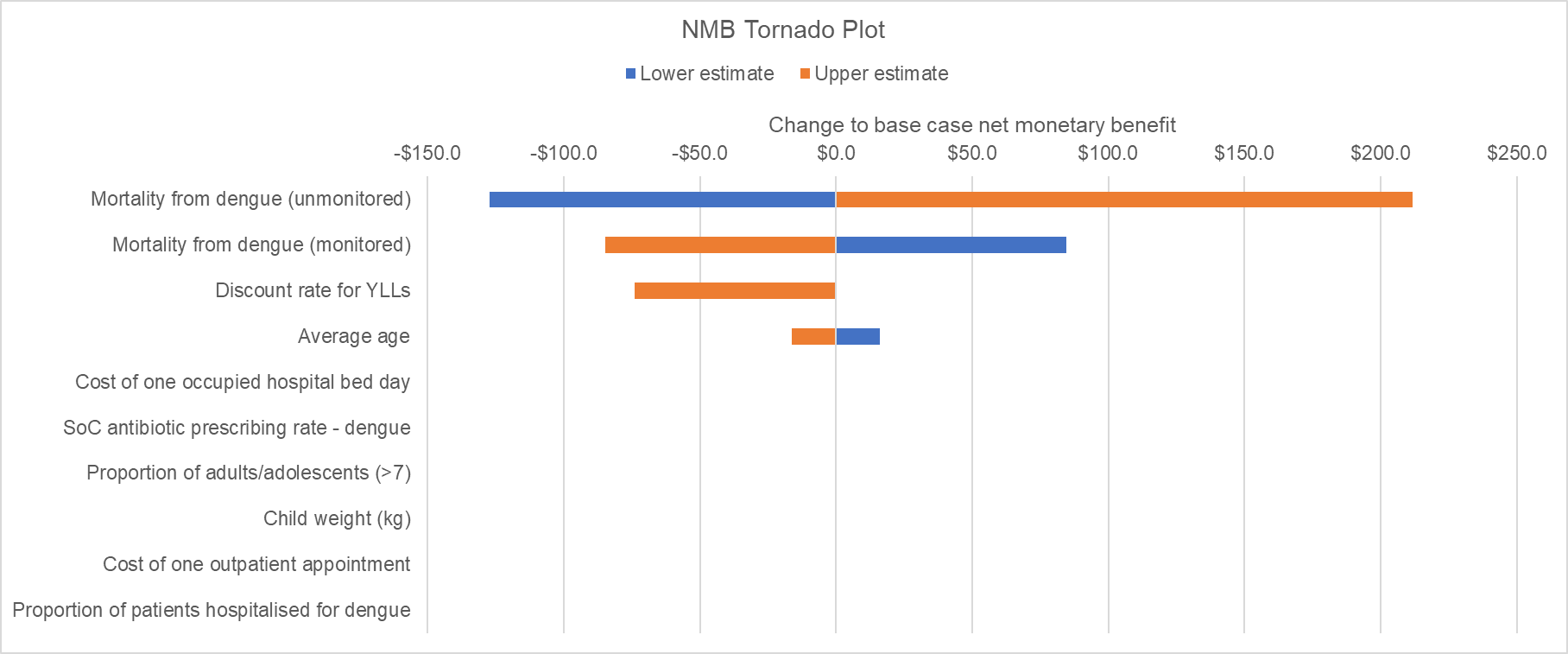


Abbreviation: YLLs, years lost of life.

Supplementary Figure 3. Deterministic sensitivity analysis tornado plot – typhoid


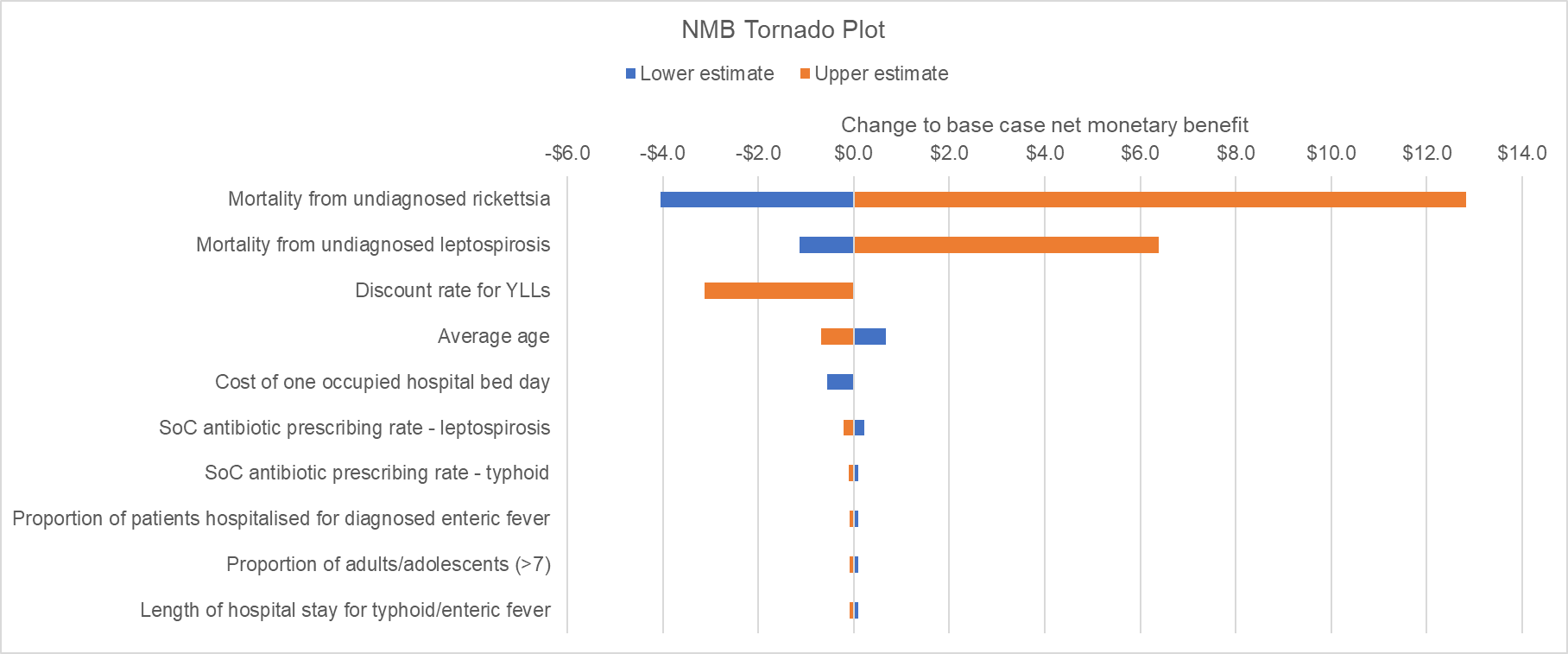


Abbreviations: SoC, standard of care; YLLs, years lost of life.

Supplementary Figure 4. Deterministic sensitivity analysis tornado plot – rickettsia


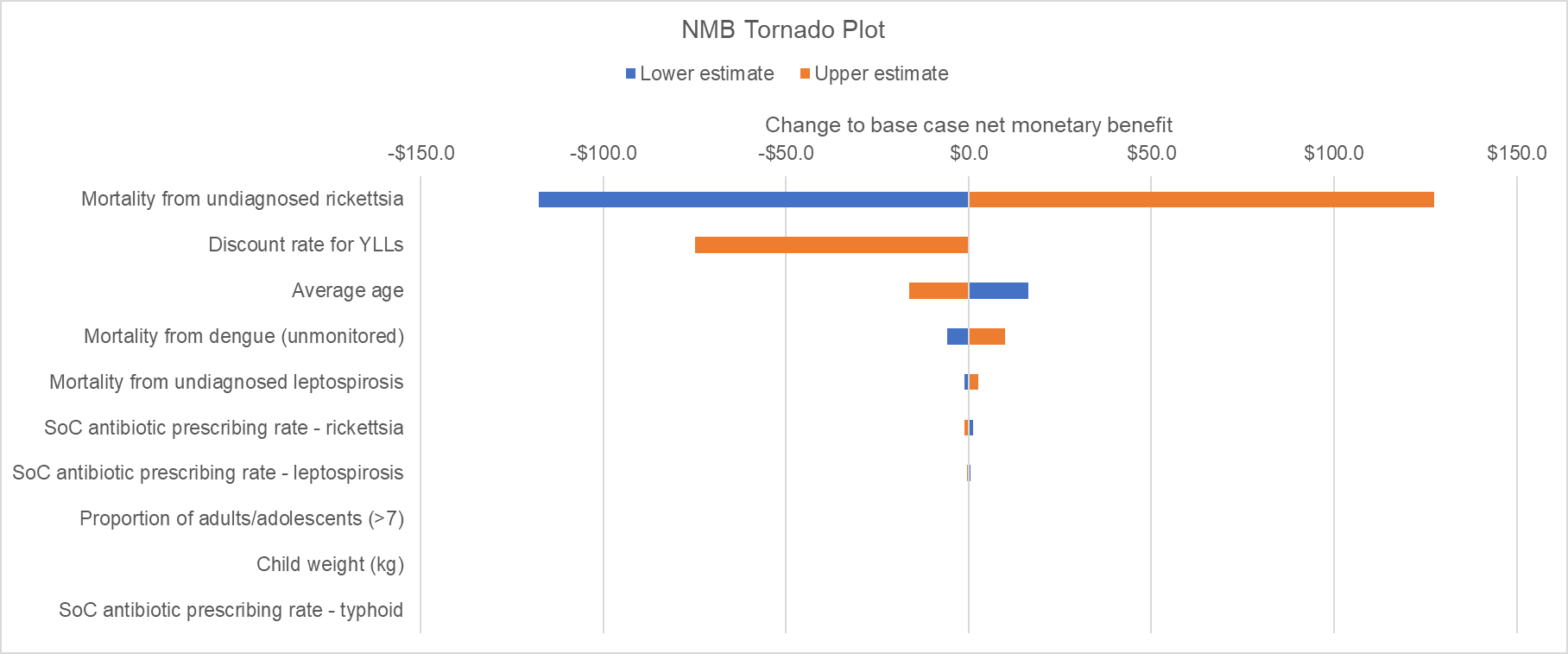


Abbreviations: SoC, standard of care; YLLs, years lost of life.

Supplementary Figure 5. Deterministic sensitivity analysis tornado plot – leptospirosis


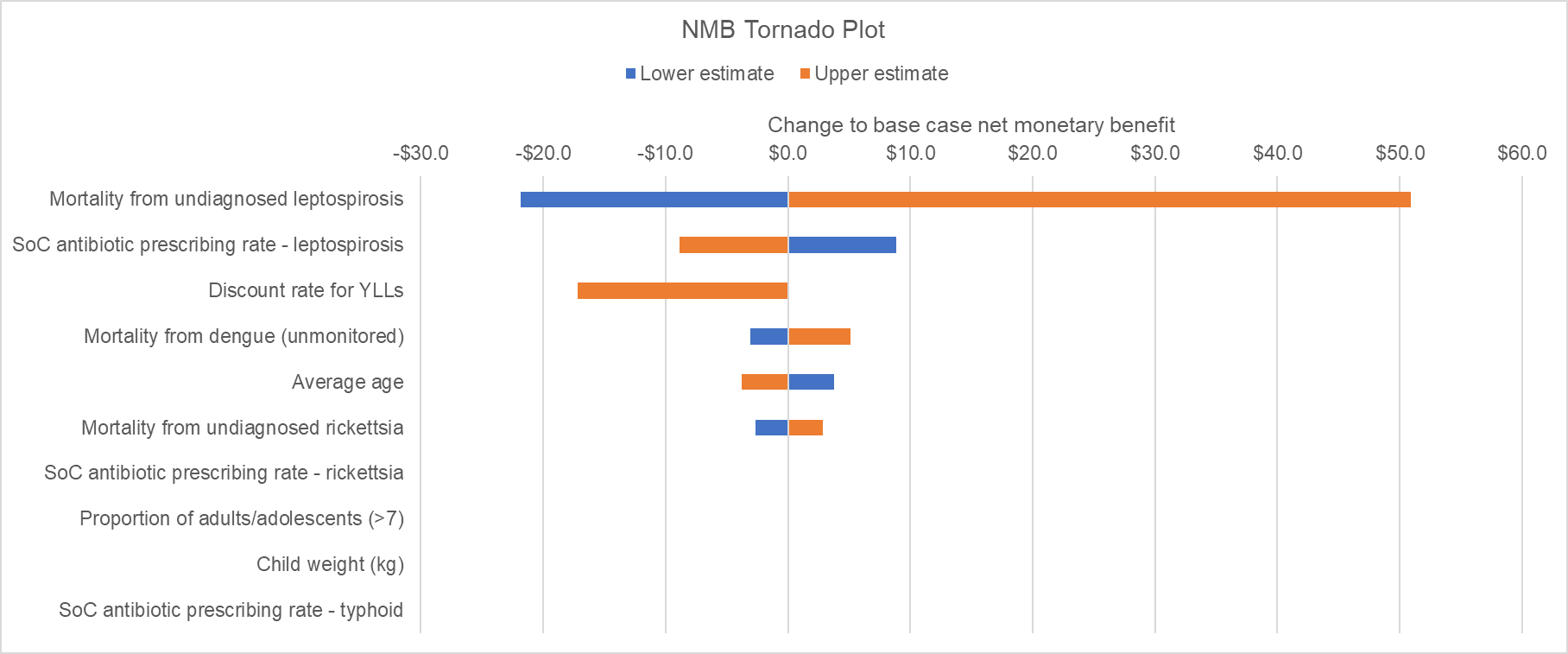


Abbreviations: SoC, standard of care; YLLs, years lost of life.

## Probabilistic Sensitivity Analyses

Supplementary Figure 6. Probabilistic sensitivity analysis results


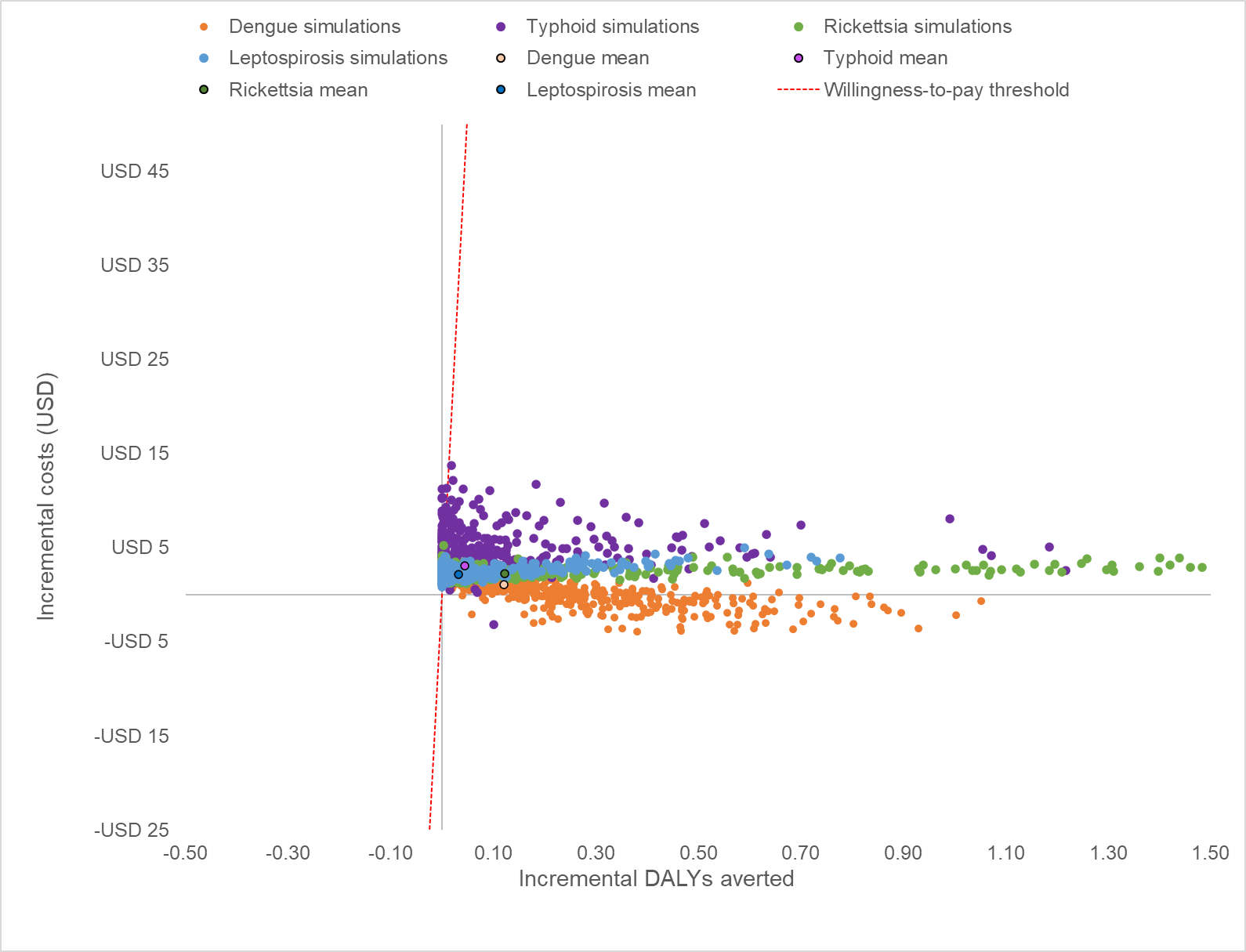


Abbreviation: DALYs, disability-adjusted life years.

## Probabilistic Epidemiological Scenario Analyses with 95% Confidence Intervals

Supplementary Figure 7. Dengue probabilistic epidemiological scenario analysis – net monetary benefit of POCT vs clinical assessment


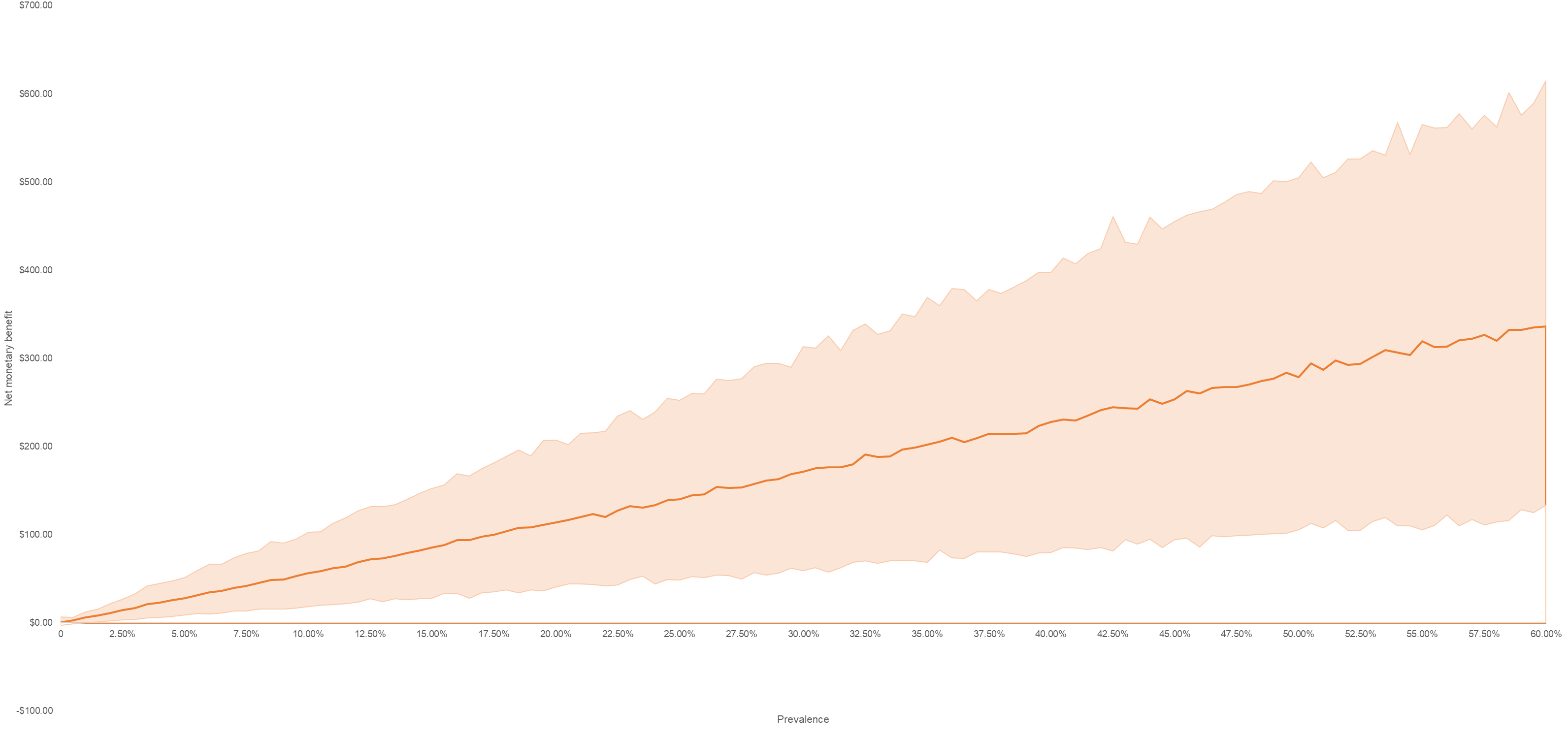


Supplementary Figure 8. Typhoid probabilistic epidemiological scenario analysis – net monetary benefit of POCT vs clinical assessment


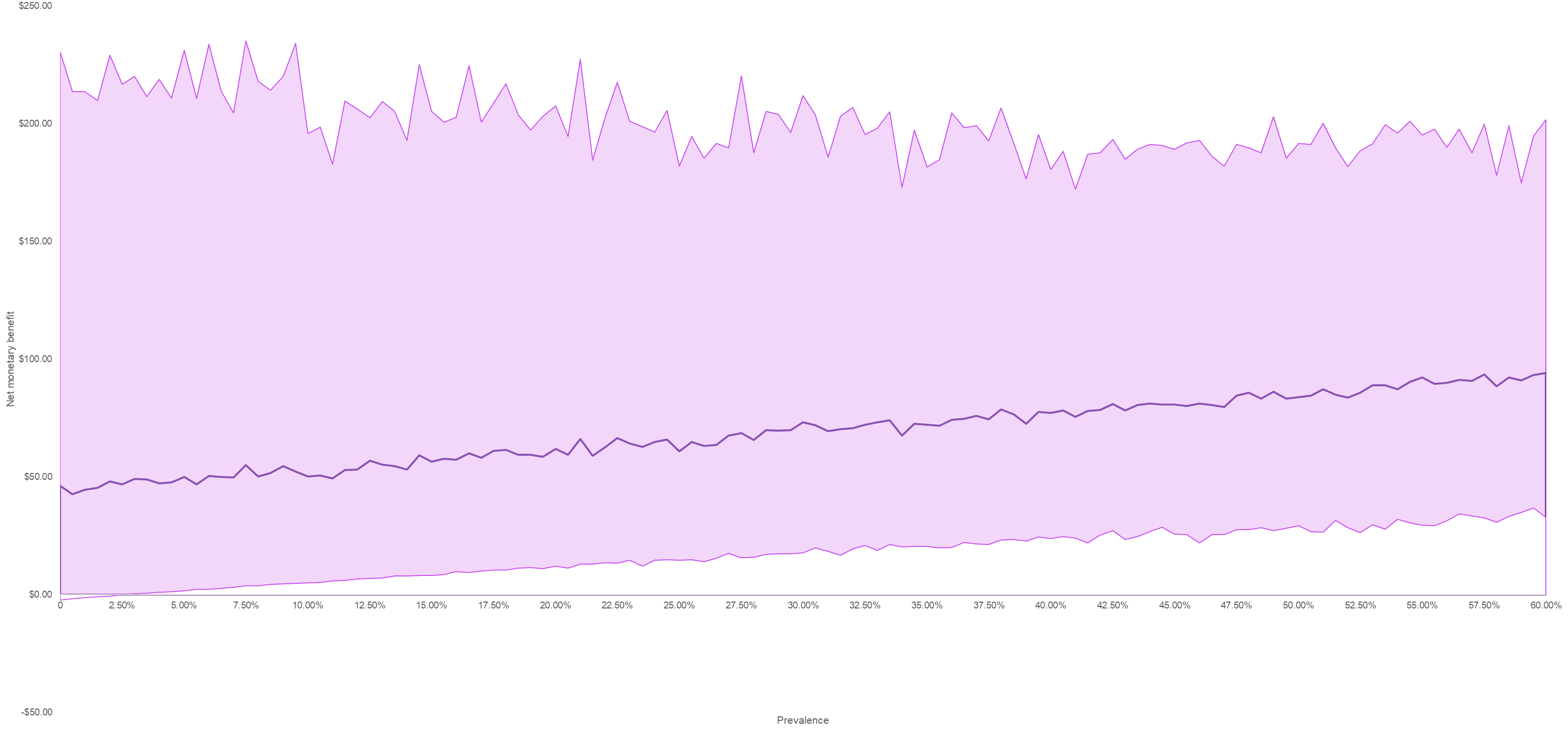


Supplementary Figure 9. Rickettsia probabilistic epidemiological scenario analysis – net monetary benefit of POCT vs clinical assessment


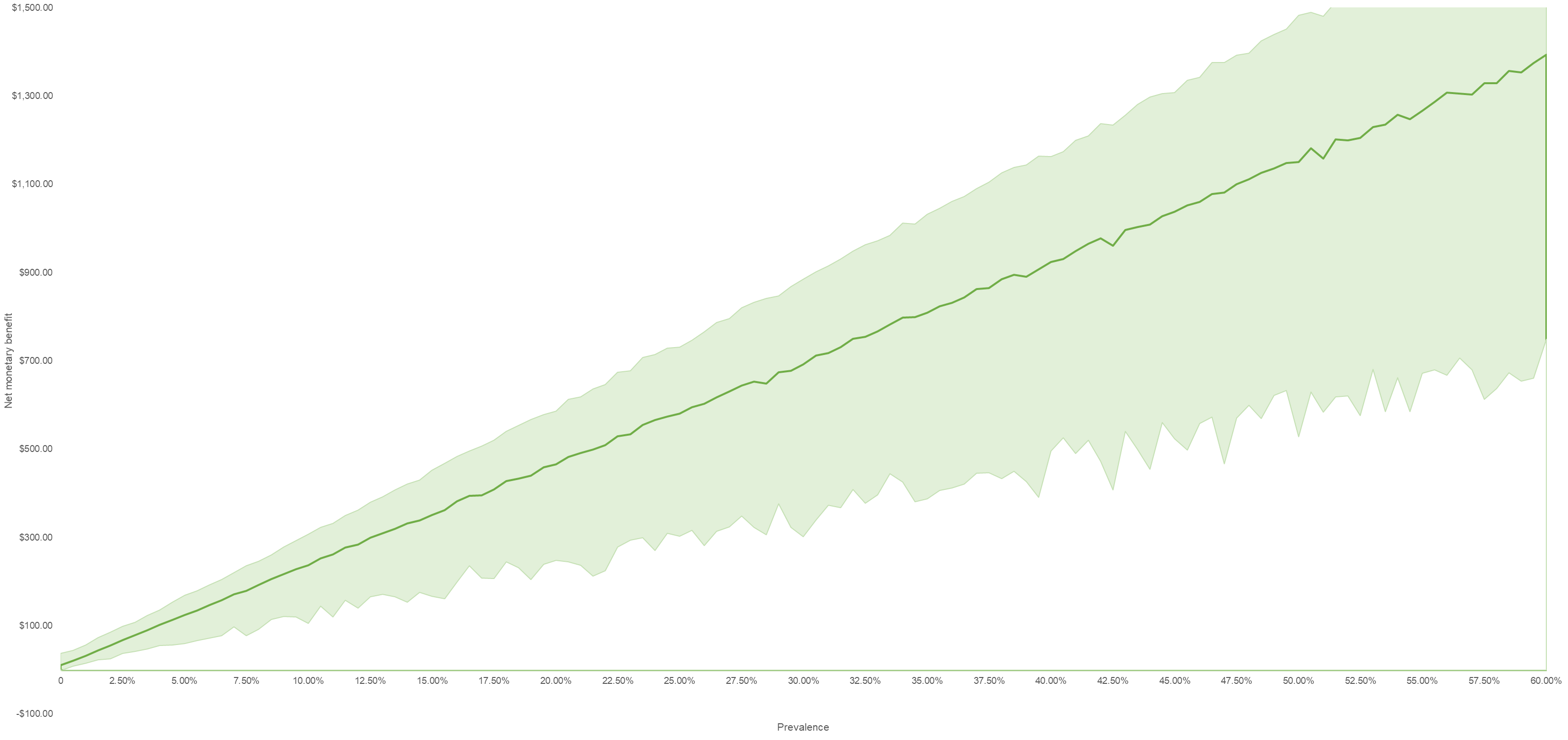


Supplementary Figure 10. Leptospirosis probabilistic epidemiological scenario analysis – net monetary benefit of POCT vs clinical assessment


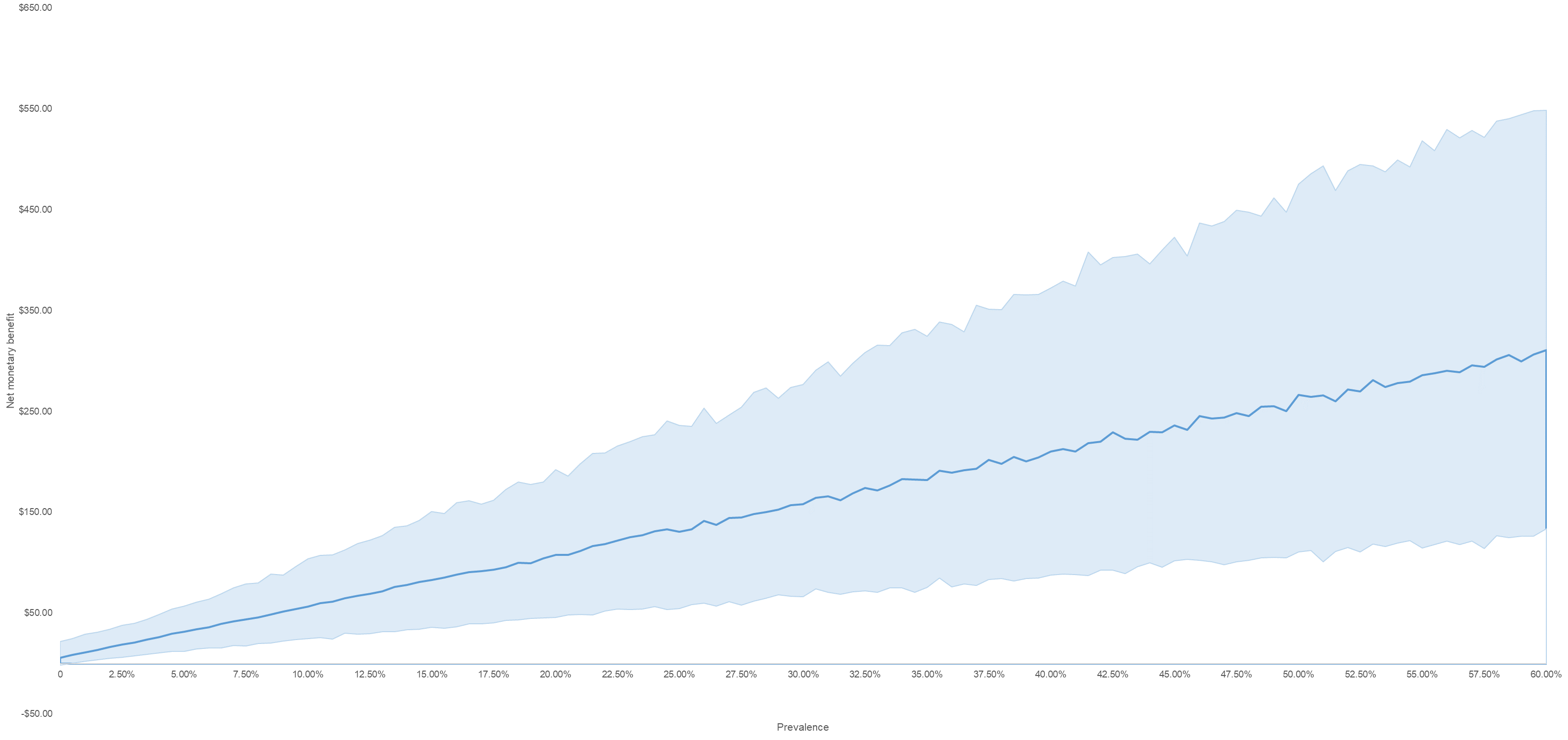


# References

1. Swe, M. Defining empirical management of acute febrile illness in Myanmar. (2022).

2. Phimda, K. *et al.* Doxycycline versus azithromycin for treatment of leptospirosis and scrub typhus. *Antimicrob Agents Chemother* **51**, 3259–3263 (2007).

3. Arjyal, A. *et al.* Gatifloxacin versus ceftriaxone for uncomplicated enteric fever in Nepal: an open-label, two-centre, randomised controlled trial. *Lancet Infect Dis* **16**, 535–545 (2016).

4. Kuehn, R. *et al.* Treatment of enteric fever (typhoid and paratyphoid fever) with cephalosporins. *Cochrane Database of Systematic Reviews* (2022) doi:10.1002/14651858.CD010452.pub2.

5. Shrestha, P. *et al.* Enumerating the economic cost of antimicrobial resistance per antibiotic consumed to inform the evaluation of interventions affecting their use. *Antimicrob Resist Infect Control* **7**, 98 (2018).
